# Miscarriage, Perceived Ostracism, and Trauma: A Preliminary Investigation

**DOI:** 10.3389/fpsyg.2021.747860

**Published:** 2022-01-27

**Authors:** Eric D. Wesselmann, Leandra Parris

**Affiliations:** ^1^Department of Psychology, Illinois State University, Normal, IL, United States; ^2^College of William & Mary, Williamsburg, VA, United States

**Keywords:** miscarriage, trauma, posttraumatic stress, ostracism, perinatal loss and grief

## Abstract

Miscarriage often is a traumatic experience with serious mental health implications. Friends and family members are often uncomfortable with and avoid discussing the topic with bereaved individuals, potentially making them feel ostracized (i.e., being ignored and excluded), contributing to their mental health concerns. We investigated the correlation between posttraumatic stress symptoms, perceived ostracism, and recalled grief intensity measures in a sample of cisgender women (*N* = 97) who have had a miscarriage. These participants were recruited using Qualtrics’s Panel Recruitment Services. Women’s perceived ostracism correlated positively with posttraumatic stress symptoms and negatively with *grief congruence* (i.e., the degree to which they felt that their miscarriage process was as satisfactory as possible, given they had to experience it). Perceived ostracism also explained additional variance in posttraumatic stress symptoms when considered alongside grief intensity measures (e.g., congruence).

## Introduction

Miscarriage often is a painful, traumatic experience for women (Kersting and Wagner, 2012). In addition to experiencing grief expressions (e.g., sadness, denial) similar to other forms of bereavement (Lasker and Toedter, 1991; Brier, 2008), women who experience miscarriage may also exhibit posttraumatic stress symptoms such as avoiding reminders and involuntarily reliving the event (Farren et al., 2016; Christiansen, 2017). Miscarriage also can lead to other mental health concerns such as anxiety-related and major depressive disorders (Swanson et al., 2009; Gold et al., 2014; Murphy et al., 2014).

There are several ways of conceptualizing and measuring the grief caused by miscarriage and other forms of perinatal loss (e.g., stillbirth), each approach having its own focus on grief manifestations (e.g., behavioral, affective, cognitive), strengths, and limitations (Setubal et al., 2021). One approach, the Perinatal Grief Intensity Theoretical Framework/Scale (Hutti and Baker, 2020), focuses on women’s perceptions of their miscarriage experience. Specifically, the framework identifies three factors that influence the intensity of the grief experience and associated mental health concerns for women post-miscarriage. Data demonstrate the experience often is more intense for women who felt the pregnancy was more “real” to them (*reality* factor), who did not feel they could confront others that said hurtful or dismissive things about the miscarriage (*confrontation* factor), and/or did not feel the overall process of the miscarriage was handled as well as could be expected (*congruence* factor; Hutti et al., 1998, 2013, 2014, 2018). Scores on these factors predict posttraumatic stress symptoms (Hutti et al., 2014), as well as mental health outcomes that co-occur with posttraumatic stress, such as anxiety and depression symptoms (Hutti and Baker, 2020). Given the mental health implications, it is imperative that researchers and clinicians understand the diverse social factors that influence women’s miscarriage experiences, grief, and trauma symptoms.

### Ostracism: A Contributing Factor to Miscarriage-Related Traumatic Stress?

In addition to typical concerns with negotiating grief, miscarriage may also involve feeling socially isolated from friends, family, and co-workers (Herz, 1984; Panuthos and Romeo, 1984). For example, qualitative data suggest that individuals who have experienced miscarriage or other forms of perinatal loss can sometimes feel like others do not acknowledge their loss (McCreight, 2004), and other research has found that these individuals can report feelings of loneliness, isolation, and being devalued (i.e., Potvin et al., 1989). One possibility is that people may feel uncomfortable talking to the bereaved individual about the loss and thus become less emotionally available or avoid the topic altogether (Harmon et al., 1984; Stroebe et al., 2013). Some individuals may minimize the bereaved person’s grief or not recognize their loss at all (Renner et al., 2000). In extreme circumstances, individuals may withdraw from the bereaved individuals entirely, as reported by bereaved individuals in other contexts (e.g., Breen and O’Connor, 2011).

Bereaved individuals who experience this lack of social support likely feel *ostracized* – a form of social exclusion characterized by feeling *ignored* or *invisible* in relationships (Williams, 2009). Ostracized individuals experience various negative psychological outcomes, such as emotional pain, feelings of being devalued, and threats to basic psychological needs (e.g., belonging, self-esteem). These outcomes occur even when the ostracism is innocuous, brief, or ambiguous, such as when someone does not receive expected text messages, social media feedback, or eye contact (Williams, 2009; Wolf et al., 2015). Additionally, various studies demonstrate that the negative effects evoked by ostracism can be re-experienced when individuals reflect on the event, even if several years have passed since the initial event (e.g., Chen et al., 2008).

Germane to the current topic, research also suggests that feeling ostracized can prolong distress (e.g., posttraumatic stress symptoms) in individuals who have experienced various forms of trauma. For example, one study (Wesselmann et al., 2018) demonstrated that perceived ostracism predicted posttraumatic stress symptoms in a sample of military veterans beyond self-reported combat stressors and perceived social support. Another study (Nietlisbach and Maercker, 2009) demonstrated that self-identified trauma survivors (e.g., accidents, natural disasters, and sexual assault) experienced significantly more short-term stress symptoms after being ostracized than control participants. Though only marginally significant, ostracized survivors reported experiencing more anxiety and psychoticism symptoms than included survivors. Time since the initial trauma did not seem to matter as the average was 9.4 years, ranging from 1 to 27 years. Given these two studies, perceived ostracism likely would have a similar effect on individuals who have experienced other forms of trauma, such as miscarriage.

### Current Research

To our knowledge, there has yet to be a systematic study of the degree to which individuals who have experienced miscarriage feel ostracized. However, extant literature supports a potential connection. Some research has found gender differences in the grieving process (Kersting and Wagner, 2012); as such, we focus our current study on cisgender women to avoid gender and sex as potential confounds. First, we hypothesized that women’s recalled grief intensity will correlate with posttraumatic stress symptoms, replicating past research (e.g., Hutti et al., 1998). Specifically, the *reality* factor will exhibit a positive correlation, and the *confrontation* and *congruence* factors will have negative correlations (Hypothesis 1).

Next, we examined our theorized links between perceived grief intensity, posttraumatic stress symptoms, and ostracism. We expected that women’s perceived ostracism will correlate positively with recalled grief intensity. Specifically, the *confrontation* and *congruence* factors will have negative correlations; these two factors explicitly deal with interpersonal interactions within the context of a miscarriage experience (Hypothesis 2a). We had no hypothesis for the *reality* factor because this factor does not pertain to interpersonal interactions. We also hypothesized that women’s perceived ostracism will correlate positively with posttraumatic stress symptoms (Hypothesis 2b). Such a correlation would replicate past research investigating ostracism with other forms of trauma (e.g., Wesselmann et al., 2018). Finally, because this study is a preliminary investigation, we chose to explore if perceived ostracism predicts incremental variance beyond grief intensity factors (Research question 1a) and the relative importance of each individual predictor (Research question 1b).

## Method

### Participants

This study was approved by the IRB at Illinois State University. We recruited participants using Qualtrics’s Panel Recruitment Services, which offers researchers the opportunity to recruit a sample of participants from their survey respondent pool that meets specified selection criteria. We specifically requested cisgender women, 18 years of age or older, who report living in the U.S. and had experienced at least one miscarriage. Further, we provided two attention checks throughout the survey (e.g., “This is an attention check: Please select “2” for this question.”). Qualtrics removed any participant who did not pass these two checks from the final dataset. After conducting a pilot study (∼20 participants), Qualtrics determined the median completion time was 8 min, so they created a speeding check to filter out participants who completed the study in under 4 min. This is standard practice for Qualtrics panels. Upon completion, participants received debriefing information and links to online mental health resources. Qualtrics then compensated participants with redeemable vouchers for various products, per their standard panel service recruiting procedures.

Ninety-seven women from 18 to 87 years of age (*M* = 51.00, *SD* = 18.34) with an average of 1.54 miscarriages (*Mode* = 1, *SD* = 0.85, *Min* = 1, *Max* = 5) participated. Most participants identified as White/Caucasian (79.8%), with 3.4% as Asian-American/Asian Descent, 8.4% Black/African American, 8.4% Hispanic/Latina, 2.5% Native American/American Indian, 2.5% Bi-/Multi-Racial, and 3.4% unspecified. Most participants (52.6%) were in long-term relationships (married/legal partnerships), with 23.7% divorced/separated, 15.5% single, and 8.2% widowed.

### Procedure and Measures

After providing consent to participate, participants were given a quality check commonly used in Qualtrics Panel studies. This question asked participants if they “commit to providing your thoughtful and honest answers in this survey?” Participants can choose: Yes, No, or I can’t commit either way. Only participants who chose “Yes” could continue. Then, participants completed basic demographics, which included the gender and miscarriage screener items. Additionally, we asked how many miscarriages they have had and how long ago (in months) was their *most recent* miscarriage. Participants meeting our criteria continued on with our measures; participants who did not indicate they had had at least one miscarriage were redirected to a different study.

#### Recalled Grief Intensity

We used the *Perinatal Grief Intensity* scale (Hutti et al., 1998) to measure participants recalled grief experiences. The measure has 14 items assessing three separate factors: *reality* (6 items, e.g., “I felt I had lost my son or my daughter, not just my pregnancy.”, Cronbach’s α = 0.87), *confront* (4 items, e.g., “In the first hours and days after my miscarriage, if people said or did things that made me feel bad, I was able to ask them to stop.”, Cronbach’s α = 0.88), and *congruence* (4 items, e.g., “During and after my miscarriage, I was satisfied with the way my miscarriage experience unfolded, given I had to go through it.”, Cronbach’s α = 0.85). Participants answered based upon their *most recent* miscarriage, using a 7-point scale (1-Strongly Disagree, 7-Strongly Agree). We averaged the items for each factor, such that higher scores indicated higher average endorsement.

#### Perceived Ostracism

Participants indicated how much they perceived being ostracized in their daily lives because of their *most recent* miscarriage, on a 10-item measure using a 7-point scale (1-Hardly Ever; 7-Almost Always; e.g., “Because of my miscarriage, I feel ignored” and “Because of my miscarriage, others treat me as if I’m in solitary confinement”; Cronbach’s α = 0.97). This measure has been used in a *general* format to examine the negative impact of perceived ostracism in adolescent (Gilman et al., 2013) and military samples (Wesselmann et al., 2018). We scored and averaged the items such that higher scores indicated higher perceived ostracism.

#### Posttraumatic Stress Symptoms

We used the PTSD Checklist for DSM-5 (PCL-5; Weathers et al., 2013) to assess participants’ self-reported posttraumatic stress symptoms. Participants answered 20 items based on the DSM-5 diagnostic criteria, rating the degree to which they had experienced each symptom within the past month (1-Not At All, 5-Extremely; Cronbach’s α = 0.97). We instructed participants to answer each question regarding their *most recent* miscarriage, adding reference to the miscarriage whenever possible in the items (e.g., ‘‘In the past month, how often were you bothered by repeated, disturbing dreams of the miscarriage.’’). We scored and averaged the items such that higher scores indicated higher reported posttraumatic stress symptoms.^1^

### Statistical Analyses

There were no missing data in our predictor or outcome measures. Participants had a wide range of time (in months) since their most recent miscarriage (2 – 816 mo.; *M* = 243, *SD* = 212.44; converted to years as *M* = 20.25, *SD* = 17.70). Because grief effects can decrease over time (Cuisinier et al., 1993), we used this as a control variable in all analyses. We examined Hypotheses 1 and 2 using bivariate partial correlations. We examined Research Question 1a using multiple regression analyses. A complimentary approach involves examining the relative importance of each predictor compared to the others in the model (Tonidandel and LeBreton, 2011; 2015). Thus, we examined Research Question 1b using a relative weights analysis (Johnson, 2000) to determine the relative contributions of the three grief intensity factors and perceived ostracism on posttraumatic stress symptoms. This analysis examined each predictor’s relative importance accounting for both the predictor’s direct effect and its effect when combined with the other predictors in the model (Johnson and LeBreton, 2004). The analysis was implemented using RWA-Web (Tonidandel and LeBreton, 2015). We calculated rescaled relative weights by dividing each relative weight by the R of the model.

## Results

Hypothesis 1 focused on replicating correlations found in previous research (Table 1). As expected, we found that two of the three recalled grief intensity factors correlated significantly with symptoms: the *more* that participants recalled being able to confront people about problematic interactions, and the *more* they recalled their miscarriage experience corresponding with their desire of care, the fewer trauma symptoms they reported (*rs* = −0.26 and −0.54, *p*s ≤ 0.010). We found a marginally significant positive correlation between reality and trauma symptoms (*r* = 0.19, *p* = 0.066).

**TABLE 1:** Descriptive statistics, inter-correlation matrix, and scale reliabilities.

	*M*	*SD*	Range	1	2	3	4	5	6
1. # of Miscarriages	1.54	0.85	4						
2. Grief: Reality	5.14	1.61	6	0.12	(*0.87*)				
3. Grief: Confrontation	4.36	1.68	6	–0.05	–0.05	(*0.88*)			
4. Grief: Congruence	4.40	1.56	6	–0.09	–0.16	0.56^∗∗^	(*0.85*)		
5. Posttraumatic stress symptoms	2.54	1.15	4	0.08	0.19^†^	−0.26^∗^	–0.54^∗∗^	(*0.97*)	
6. Perceived ostracism	2.07	1.46	6	0.20^†^	–0.09	–0.04	–0.34^∗∗^	0.54^∗∗^	(*0.97*)

*^†^p < 0.07, ^∗^p < 0.01, ^∗∗^p ≤ 0.001; correlations are partial with length of time since last miscarriage as control. Italicized numbers are the Cronbach’s α for each measure.*

Hypothesis 2a focused on the correlations between women’s perceived ostracism and the three factors of their recalled grief intensity. Perceived ostracism only correlated significantly with grief congruence, such that the more participants recalled their grief experience as being congruent with their expectations, the less they felt ostracized (*r* = −0.34, *p* = 0.001). Hypothesis 2b focused on the correlation between women’s perceived ostracism and their posttraumatic stress symptoms, for which we found a significant positive correlation (*r* = 0.54, *p* < 0.001).

For Research Question 1a, we explored if perceived ostracism predicts incremental variance beyond grief intensity factors. Perceived ostracism explained additional variance in posttraumatic stress symptoms beyond the three grief intensity factors: Δ*R*^2^ = 0.12, *F* (1, 91) = 26.99, *p* < 0.001. The full results are presented in Table 2. For Research Question 1b we examined the relative importance of each predictor compared to the others in the model and found that perceived ostracism (40.99%; raw weight = 0.24) explained the most variance, followed by the grief congruence factor (26.10%; raw weight = 0.15), length of time since the most recent miscarriage (23.77%; raw weight = 0.14), grief confront (5.86%; raw weight = 0.03), and grief reality (2.27%, raw weight = 0.02).

**TABLE 2:** Incremental variance explained by perceived ostracism over grief components.

Outcome: Posttraumatic stress symptoms				
Step	Model summary	Predictor variable	B	Δ*R*^2^
1	*R*^2^ = 0.23, *F* (1,95) = 28.74***	Time since Last Miscarriage	–0.48^∗∗∗^	
2	*R*^2^ = 0.47, *F* (4,92) = 20.11***	Time since Last Miscarriage	–0.41^∗∗∗^	
		Reality	0.09	
		Confrontation	0.05	
		Congruence	–0.49^∗∗∗^	0.23^∗∗∗^
3	*R*^2^ = 0.59, *F* (5,91) = 26.03***	Time since Last Miscarriage	–0.28^∗∗∗^	
		Reality	0.15^∗^	
		Confrontation	–0.04	
		Congruence	–0.30^∗∗^	
		Perceived Ostracism	0.41^∗∗∗^	0.12^∗∗∗^

*^∗^p < 0.050, ^∗∗^p = 0.001, ^∗∗∗^p < 0.001. Betas are standardized.*

## Discussion

We provide preliminary empirical evidence for a connection between perceived ostracism and women’s miscarriage-related posttraumatic stress symptoms. Specifically, women who perceived more ostracism reported higher levels of posttraumatic stress symptoms, with a large effect size (Murphy and Myors, 2004). Further, it explained additional variance when considered alongside established grief intensity factors, this time with a medium effect size. Indeed, it predicted the most amount of variance comparatively. Thus, our data suggest that researchers interested in miscarriage-related grief and posttraumatic stress should consider how perceived ostracism may contribute to—and prolong—these processes. Such connections would have important implications for mental health treatment plans used with women whose presenting concerns involve miscarriage.

These data provide an important first step but there are several factors limiting the generalizability of our results. We used a cross-sectional survey approach and are thus limited in any causal interpretations of our data. Additionally, we did not specify that participants had to be within a set timeframe post-miscarriage; for some participants it may have been a matter of a few months, and for others it may have been several years. However, we do not believe this limitation presents an alternative explanation for our results. Previous research has established that the emotional pain of ostracism can be re-experienced long after the initial event occurred (e.g., Chen et al., 2008). Even though grief reactions tend to dissipate over time (Cuisinier et al., 1993) some research has found that negative effects can linger for several years (Murphy et al., 2014). Indeed, some qualitative studies found that people report experiencing the pain of losing a child (either through miscarriage or to a terminal illness post-birth) for several decades (McClowry et al., 1987; Hazen, 2003). Some bereavement experts have argued that for many people the pain may never go away completely (e.g., Corr, 1998-99). Trauma experts have argued that there is no specific timeframe for when trauma can no longer manifest and it can become a chronic condition that impacts individuals over the lifespan (Van Der Kolk, 2015; National Institute of Mental Health, 2019). This may be especially true for individuals who were not allowed to mourn their loss (Hazen, 2003). Finally, any dissipation occurring in our sample would have made it more difficult for us to find any relations between our measures, yet we still did. In our study, length of time was only the third largest predictor of variance in a relative weights analysis, whereas perceived ostracism and grief congruence were the first and second largest, respectively. Regardless, future research should replicate these findings using multi-wave longitudinal survey designs with larger samples to examine the temporal structure of grief intensity, perceived ostracism, and posttraumatic stress symptoms.

There are other demographic factors which we did not assess that may also influence our variables of interest, such as education, socioeconomic status, number of living children, gestational age when the miscarriage occurred, or reason for the miscarriage. People may be less willing to support a bereaved mother if the miscarriage occurred early in gestation or if she has other living children. Future research should investigate the degree to which these factors influence the connection between perceived ostracism and trauma symptoms. Additionally, we did not specifically screen participants for clinical diagnoses of posttraumatic stress disorder. Rather we simply included all participants’ data using the continuous nature of our outcome measure, regardless of whether it reached a clinical threshold. It is important to note that two previous studies investigating the connection between ostracism and trauma symptoms did not restrict their sampling to a clinical population either and still found consistent effects that converge with our current findings (Nietlisbach and Maercker, 2009; Wesselmann et al., 2018). Regardless, these effects may be intensified in a clinical population.

Similarly, we used the Perinatal Grief Intensity scale as a continuous variable rather than a clinical screening tool. As with the posttraumatic stress measure, it is likely that the effect sizes would be magnified for individuals who fit the criteria for a clinical diagnosis of intense grief responses. As previously mentioned, there are multiple grief measures used in research on perinatal loss, each with their individual foci, strengths, and limitations (Setubal et al., 2021). Future research should investigate how these measures relate to perceived ostracism, posttraumatic stress, and other grief-related phenomena. For example, the Perinatal Grief Scale is another common measure (Toedter et al., 1988). Data suggest that the measure we used is comparable with this scale (Hutti et al., 2017; Hutti and Baker, 2020), yet it would be useful to examine these relations directly as a conceptual replication. Future research should also investigate how perceived ostracism relates to the experience of *prolonged grief* – an attachment related disorder that involves threats to one’s sense of meaning, identity, and a yearning for the deceased person. Prolonged grief often co-occurs with other mental health concerns such as posttraumatic stress, depression, and anxiety, yet it is a distinct construct that researchers interested in both perinatal loss and perceived ostracism should consider (Prigerson et al., 2021).

Finally, our sample also is limited to cisgender women. We cannot generalize our findings to transgender women, or to *cis*- and transgender men, or individuals with non-binary gender identities. Previous research suggests that although men also experience grief, posttraumatic stress symptoms, and other mental health issues post-miscarriage, the processes and intensity can differ from those of women (Johnson and Baker, 2004; McCreight, 2004; Kersting and Wagner, 2012). In some cases, cultural expectations such as viewing men’s role solely as being a support partner may lead friends, family, and even medical staff to ignore their grief (Jones et al., 2019). Gender identity may complicate this process further. Individuals who hold transgender or non-binary gender identities often experience discrimination *via* purposeful misgendering, which can make them feel excluded/ignored (Wesselmann et al., 2021). If these individuals have friends, family, or medical personal who refuse to use their preferred parental terms or refuse to acknowledge their parental status, they are likely to experience additional ostracism that cisgender individuals do not.

Additionally, connecting the ostracism and miscarriage literature provides an opportunity to examine the complex nature of *disenfranchised grief*, a type of grief “persons experience when they incur a loss that is not or cannot be openly acknowledged, publicly mourned, or socially supported” (Doka, 1989, p.4). Research on disenfranchised grief suggests it is a multifaceted experience involving both interpersonal and structural factors, all of which combine to make the bereaved person feel that some aspect of their grief is invalidated, devalued or unrecognized (Corr, 1998-99). Qualitative research demonstrates that women who have had miscarriages indicate both interpersonal and system-level experiences relevant to disenfranchised grief in other bereavement contexts (Hazen, 2003; Lang et al., 2011; Mulvihill and Walsh, 2014). Based on our data, perceived ostracism seems to be an important interpersonal factor that contributes to this overall experience and should be considered by researchers interested in this construct, both within the context of miscarriage and bereavement broadly.

## Data Availability Statement

The original contributions presented in the study are publicly available. This data can be found here: https://osf.io/vkz23/.

## Ethics Statement

The studies involving human participants were reviewed and approved by Illinois State University Institutional Review Board. Written informed consent for participation was not required for this study in accordance with the national legislation and the institutional requirements.

## Author Contributions

Both authors listed have made a substantial, direct, and intellectual contribution to the work, and approved it for publication.

## Conflict of Interest

The authors declare that the research was conducted in the absence of any commercial or financial relationships that could be construed as a potential conflict of interest.

## Publisher’s Note

All claims expressed in this article are solely those of the authors and do not necessarily represent those of their affiliated organizations, or those of the publisher, the editors and the reviewers. Any product that may be evaluated in this article, or claim that may be made by its manufacturer, is not guaranteed or endorsed by the publisher.

## Footnotes

1 We collected a measure of basic need satisfaction and psychological distress as additional outcome measures. As in other research, higher perceived ostracism correlated with lower basic need satisfaction. The results for psychological distress were similar to those for posttraumatic stress symptoms, albeit with smaller effect sizes. Data available upon request.
